# Nuclear Accumulation of Stress Response mRNAs Contributes to the Neurodegeneration Caused by Fragile X Premutation rCGG Repeats

**DOI:** 10.1371/journal.pgen.1002102

**Published:** 2011-06-02

**Authors:** Abrar Qurashi, Wendi Li, Jian-Ying Zhou, Junmin Peng, Peng Jin

**Affiliations:** 1 Department of Human Genetics, Emory University School of Medicine, Atlanta, Georgia, United States of America; 2 Center for Neurodegenerative Disease, Emory University School of Medicine, Atlanta, Georgia, United States of America; 3 Emory Proteomics Service Center, Emory University School of Medicine, Atlanta, Georgia, United States of America; University of Minnesota, United States of America

## Abstract

Fragile X–associated tremor/ataxia syndrome (FXTAS) is a neurodegenerative disorder seen in Fragile X premutation carriers. Previous studies found that Fragile X rCGG repeats are sufficient to cause neurodegeneration and that the rCGG repeat-binding proteins Pur α and hnRNP A2/B1 can modulate rCGG–mediated neuronal toxicity. To explore the role of Pur α in rCGG–mediated neurodegeneration further, we took a proteomic approach and identified more than 100 proteins that interact with Pur α. Of particular interest is Rm62, the *Drosophila* ortholog of p68 RNA helicase, which could modulate rCGG–mediated neurodegeneration. Here we show that rCGG repeats decreased the expression of Rm62 posttranscriptionally, leading to the nuclear accumulation of *Hsp70* transcript, as well as additional mRNAs involved in stress and immune responses. Together these findings suggest that abnormal nuclear accumulation of these mRNAs, likely as a result of impaired nuclear export, could contribute to FXTAS pathogenesis.

## Author Summary

Fragile X–associated tremor/ataxia syndrome (FXTAS) is an adult-onset neurodegenerative disorder that usually affects males over 50 years of age, and FXTAS patients are carriers of fragile X premutation alleles. Using a FXTAS *Drosophila* model, we previously showed that fragile X premutation rCGG repeats alone could cause neurodegeneration. Pur α and hnRNP A2/B1 were identified as specific premutation rCGG repeat-binding proteins (RBPs) that could bind and modulate fragile X premutation rCGG–mediated neuronal degeneration. Here, through systematic proteomic, genetic, and microarray analyses, we show that the nuclear accumulation of select mRNAs caused by fragile X premutation rCGG repeats may contribute to FXTAS pathogenesis, and the mechanism could be via impaired nuclear export due to the decreased levels of Rm62 seen upon fragile X premutation rCGG expression.

## Introduction

Fragile X syndrome (FXS) is usually caused by expansion of the CGG trinucleotide repeat in the 5′ untranslated region (5′ UTR) of the Fragile X Mental Retardation 1 (*FMR1*) gene [1]. Whereas normal individuals generally possess between five and 54 repeats, fully affected individuals have more than 200 CGG repeats on what are referred to as full mutation alleles [2]. Premutation alleles (55–200 CGG repeats) of the *FMR1* gene are known to contribute to the Fragile X phenotype through genetic instability, and they can expand into the full mutation during germline transmission [3]. Within the last decade, Fragile X-associated Tremor/Ataxia Syndrome (FXTAS), a late-onset neurodegenerative disorder, has been recognized mainly among many male premutation carriers in or beyond their fifth decade of life [4], and FXTAS is distinct from the neurodevelopmental disorder, FXS.

The most common clinical feature of FXTAS is a progressive action tremor with ataxia. More advanced or severe cases may show a progressive cognitive decline that ranges from executive and memory deficits to dementia [5]. Patients may also present with common psychiatric symptoms, such as increased anxiety, mood liability, and depression [6], [7]. Magnetic resonance imaging (MRI) of adult male patients affected with FXTAS demonstrated mild to moderate global brain atrophy, most common in the fontal and parietal regions, as well as the pons and the cerebellum [8]. Nearly all autopsy studies on the brains of symptomatic premutation carriers show degeneration in the cerebellum, which includes Purkinje neuronal cell loss, Bergman gliosis, spongiosis of the deep cerebellar white matter, and swollen axons [9], [10]. The major neuropathological hallmark and postmortem criterion for definitive FXTAS is eosinophilic, ubiquitin-positive intranuclear inclusions broadly distributed throughout the brain in neurons, astrocytes, and in the spinal column [9].

For the past several years, the hunt has been on to uncover the molecular basis of FXTAS. One unique molecular signature of the fragile X premutation allele is that the level of *FMR1* mRNA is significantly elevated, while the FMR1 protein (FMRP) remains relatively unchanged in cells from premutation carriers [11], [12]. Given that the neurodegenerative phenotype of FXTAS is associated specifically with premutation carriers, but not with the full mutation, FMRP deficiency per se is likely not the culprit behind FXTAS [4]. Instead, the neurodegenerative phenotypes associated with FXTAS are suspected of being caused by a gain of function in fragile X premutation rCGG repeat RNAs [3], [13]. The hypothesis is that overproduced rCGG repeats in FXTAS sequester specific RNA-binding proteins and reduce their ability to perform their normal cellular functions, thereby contributing significantly to the pathology of this disorder. The presence of *FMR1* mRNA in inclusions found in the brains of FXTAS patients, as well as the formation of similar inclusions upon ectopic expression of rCGG repeats in model systems, have provided strong support for this hypothesis [13]–[16].

Two RNA-binding proteins, Pur α and hnRNP A2/B1, are known to bind rCGG repeats specifically in both mammalian and *Drosophila* brains [17], [18]. Both Pur α and hnRNP A2/B1 are found to be present in the inclusions of FXTAS brain tissues. Furthermore, overexpression of either Pur α or hnRNP A2/B1 can alleviate neurodegeneration in the fly model of FXTAS [17], [18]. In particular, Pur α knock-out mice appeared normal at birth, but developed severe tremor and spontaneous seizures at two weeks of age due to drastically reduced numbers of neurons in regions of the hippocampus and cerebellum, suggesting that the depletion of Pur α alone could lead to ataxia [19].

In this study, to further investigate the role of Pur α in rCGG-mediated neurodegeneration, we took a proteomic approach to identify the proteins that interact with Pur α. Over 100 proteins, including several known interactors, such as Fmrp, were found to interact with Pur α *in vitro*. To evaluate the physiological role(s) of Pur α-interacting proteins in rCGG-mediated neuronal toxicity, we further tested their genetic interactions with rCGG repeats using our FXTAS fly model and identified several interactors of Pur α that could genetically modulate the toxicity caused by rCGG repeats. Among these, Rm62, the *Drosophila* ortholog of the p68 RNA helicase, was of particular interest. Rm62, physically interacting with Pur α, could modulate rCGG-mediated neurodegeneration, and biochemically, fragile X rCGG repeats could decrease the expression of Rm62 posttranscriptionally, leading to the accumulation of *Hsp70* transcript, a previously identified target of Rm62, in the nucleus. Further microarray analyses revealed the nuclear accumulation of additional mRNAs involved in stress and immune responses in fragile X premutation flies. These findings suggest an unexpected nuclear accumulation of specific mRNAs caused by fragile X premutation rCGG repeats and point to likely deficits in the nuclear export of specific mRNA as a possible cause of the compromised stress response in neurons expressing rCGG repeats, which would predispose them to neuronal apoptosis.

## Results

### Identification of a Pur α interactome

**Figure 1:**
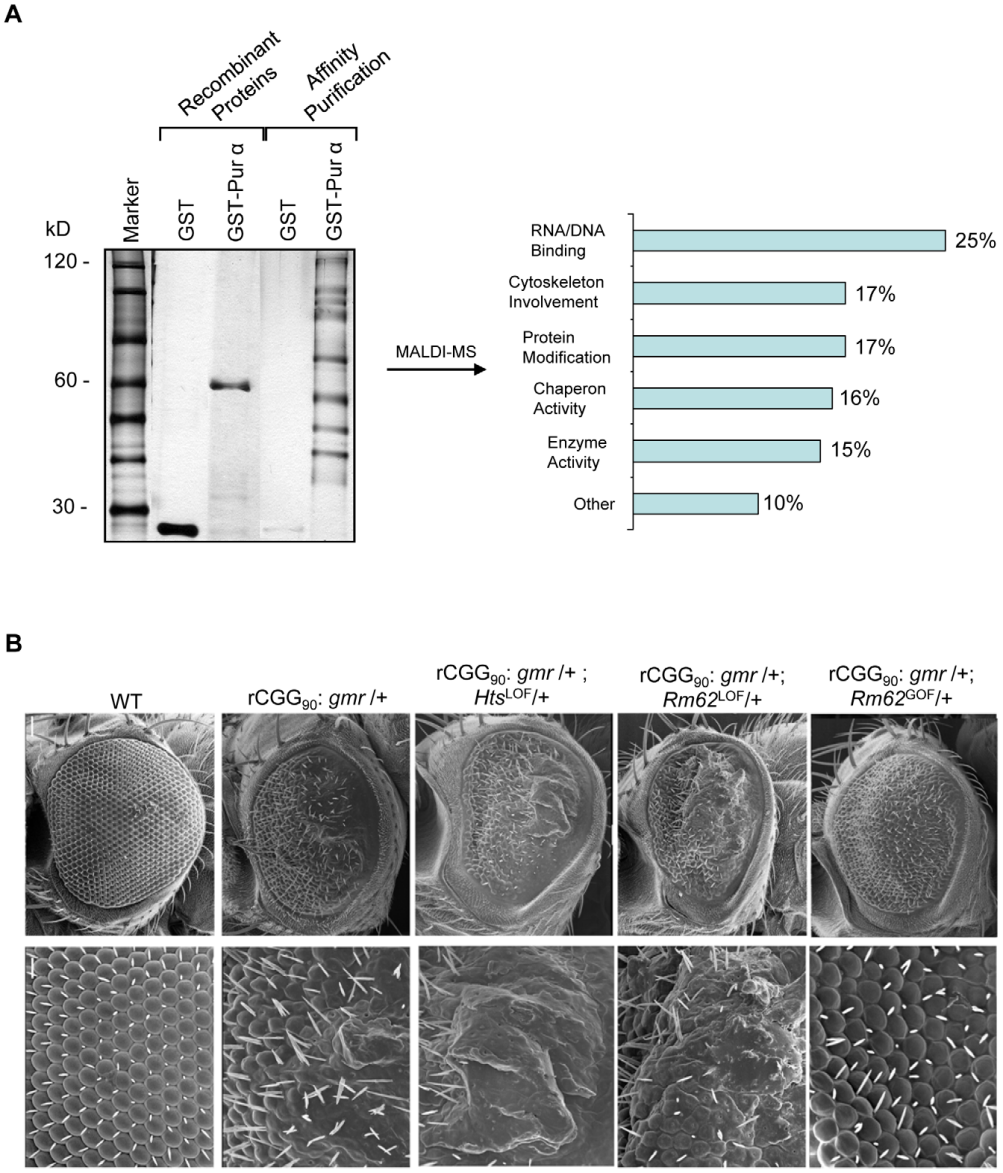
Identification of Pur α-interacting proteins. A. Silver-staining gel with distinct bands for recombinant proteins and affinity-purified proteins. The captured proteins were further analyzed by mass spectrometry, and distinct classes of proteins were identified. B. *Rm62* and *Hts* mutants enhance rCGG-mediated neurodegeneration in fly. Column 1: wild-type fly; Column 2: fly expressing (CGG)_90_-EGFP only; Column 3: fly expressing (CGG)_90_-EGFP in the heterozygous background of *Hts* ^01103^ Loss-of-Function (LOF) mutation; Column 4: fly expressing CGG_90_-EGFP in the heterozygous background of *Rm62* ^01084^ Loss-of-Function (LOF) mutation; Column 5: fly expressing CGG_90_-EGFP in the heterozygous background of *Rm62* ^(3)3607^ overexpression (Gain-of-Function (GOF). SEM eye images are shown.

Our previous studies have shown that the neuronal apoptosis caused by fragile X premutation rCGG repeats arose at least in part as a consequence of Pur α sequestration [17]. We therefore hypothesized that genetic modifiers of rCGG-mediated neurodegeneration should be enriched among Pur α-interacting proteins. To test this idea, we took a proteomic approach to identify a comprehensive set of Pur α-interacting proteins (Pur α interactome). GST-Pur α fusion proteins were expressed in *Escherichia coli* and purified by glutathione-sepharose resins. The resins with the bound proteins were directly used to pack affinity columns. The columns were then loaded with pre-cleared wild-type fly brain extracts, washed extensively, and eluted. The eluted proteins were resolved on an SDS gel. As indicated in Figure 1A, a gel of the indicated size was followed by the analysis of liquid chromatography (LC)-tandem mass spectrometry (LS/MS). GST alone was also used for affinity purification as a negative control (Figure 1A). Through these analyses, we identified over 100 proteins that could interact with Pur α *in vitro*. The molecular functions of the identified proteins include RNA-binding/DNA-binding, cytoskeleton network, molecular chaperone, and ubiquitin proteasome pathways (Figure 1A and Table 1). Importantly, among these proteins, we also identified Cdk5 and Fmrp, which are known to interact with Pur α biochemically, suggesting the approach we have used to identify the Pur α interactome is reliable.

**Table 1:** Pur α Interactome.

Functional Class	Identified Proteins	Mammalian Ortholog
**RNA binding/DNA binding**	Pur α	Pur α
	Rm62	p68
	Fmrp	Fmrp
	Ypsilon Schachtel	Y box protein 1
	Glorund	-
	Lark	RNA binding protein 4B
	Vasa intronicgene	SERBP1
	Aly	Tho4
	CG30122	HnRNP U-like protein 1
	SF2	SFRS1
	B52	SFRS6
	X16	SFRS7
	CG7185	
	RNA binding protein 9	Elav-like protein 2
	RNA binding protein 1	-
	U2 small nuclear riboprotein auxiliary factor	U2
	Repressor splicing factor1	-
	Serine-arginine protein 55	
	Cleavage and polyadenylation specificity factor subunit CG7185	
	RNA binding protein 1, -9	
	Histone H1	Histone
	Ribosomal protein L11, -l5, -S20, -S4, -S9,	
	CG33801; -33807; -33834, -33858, -33861, -33855, -10203, -1101, -14648, -17838, -6987	
**Chaperone/response to stress**	Hsp70 Aa, Ab, Ba, Bb, Bc, 68	Hsp70
	Heat Shock protein cognate 2, 4, 5	HspA9B
	Ubiquitin-63E	
	Roe	GrpE protein homolog 1
**Cytoskeleton involvement/structural molecules**	Tropomyosin 1, -2	Tropomyosin
	Hu li Tai Shao	Adducin 1
	Upheld	TNNT3
	Coracle	Band 4.1 like protein 3
	Actin related protein 53D	Arp
	Actin 5C, -42A, - 57B, -79B, -87E, 88F	Actin
	Myosin heavy chain 1, -2,	MHC
	Myosin Alkali light chain	-
	Lamin	-
	Paramyosin	-
	Retinoid- and fatty acid-binding glycoprotein	-
	β Tubulin 56D, -60D, -97EF, -85D	Tubulin
	α Tubulin 84D, -84B, -85^E^	Tubulin
	Ribosomal proteins	Ribosomal proteins
**Enzyme activity**	Glutathione S transferase E1, E6, S1	
	Thiolase	
	Sluggish A	
	Bellwether	ATP synthase α
	CG12163	-
	ATP synthase –α, -β	ATP synthase –α, -β
	Adenine nucleotide translocase	ADP/ATP translocase-2
	CG11198	Acetyl – CoA carboxylase 1
	CG5028	
	CG3689	
	Stoned A	
**Protein modification**	Adaptin	
	Arrestin	
	Retinoid and fatty acid binding protein	
	UbiP63E, -P5E	
	Calcium/calmodulin –dependent protein kinaseII	CamKII
	NinaC	Myosin IIIB
	Cdk5	Cdk5
	Retinal degeneration A	-

### The Pur α–interacting proteins Rm62 and Hts modulate rCGG–mediated neurodegeneration

To determine which Pur α-interacting protein(s) modulates rCGG-mediated neurodegeneration, we conducted a genetic screen based on the fragile X premutation rCGG repeat-mediated neurodegenerative eye phenotype that we observed previously [13]. The screen involved directing the expression of fragile X premutation-length rCGG repeats to the eye with the *Gmr-GAL4* driver using the *Drosophila* GAL4/UAS system. This was followed by crossing *Gmr-GAL4, UAS-(CGG)_90_-EGFP* transgenic flies with flies mutant in genes coding the Pur α-interacting proteins. We selected 17 mutant alleles for evaluation based on their availability from several stock centers (Table 2). The progenies were examined for potential suppression or enhancement of the disorganized eye phenotype compared with control rCGG flies. Through this screen, we identified two prominent enhancers that could modulate rCGG-mediated neurodegeneration, *l(3)01084* and *l(3)01103*. P-element insertion *l(3)01084* was previously characterized as an insertion within the 5′ region of the *Rm62* gene that causes lethality [20] (Figure 1B). *Rm62* is a homolog of mammalian *p68* RNA helicase, which has been implicated in transcriptional regulation, pre-mRNA splicing, RNA interference, and nucleocytoplasmic shuttling [21]–[26]. The modulation of rCGG-mediated neuronal toxicity by Rm62 was further confirmed using additional loss-of-function alleles of Rm62. Furthermore, overexpression of Rm62 could suppress the neuronal toxicity caused by fragile X premutation rCGG repeats. The other enhancer line, l(3)01103, is a mutant allele of the *hu-li tai shao* (*hts)* gene, a homolog of mammalian *Adducin,* which has been shown to play key roles during early *Drosophila* oogenesis [27] (Figure 1B).

**Table 2:** Mutant Alleles Corresponding to Pur α-Interacting Proteins Used for Genetic Screen.

Annotation	Gene	Alleles	Phenotypic effect on *gmr:CGG_90_-EGFP/+*
CG5125	Nina C	*nina C^2^, nina C^5^*	*—*
CG7107	Upheld	*up^1^*	*—*
CG4264	Hsc70	*Hsc70^403550^*	*—*
-	Lethal(2)k06416	*l(2)k06416^k06416^*	*—*
CG9325	Hu i tai shao (Hts)	*hts^01103^*	***Enhancer***
CG6944	Lamin	*Lam^04643^*	*—*
CG10279	Rm62	*Rm62^01086^, Rm62^06795^, Rm62^DG12402^, RM62^EY10915^	***Enhancer***
CG5939	Paramyosin	*Prm^10631^*	*—*
CG3612	Bellwether	*blw^1^*	*—*
CG4260	α-Adaptin	*α-Adaptin^06694^*	*—*
CG3151	RNA-binding protein 9	*Rbp9^BG02784^*	*—*
CG1417	Sluggish A	*slgA^KG07965^*	*—*
CG10686	Trailer hitch	*tral^KG08052^*	*—*
CG11064	Retinoid- and fatty acid-binding glycoprotein	*Rfabg^C204^*	*—*
CG3506	Vasa	*vas^EY07816^*	*—*
CG18069	CaMKII	*CaMKII^EY14097^*	*—*
CG3082	Lethal (2) k09913	*l(2)k09913^EY20574^*	*—*

***—***: Very Mild or No effect.

### Both Rm62 and Hts directly interact with Pur α

**Figure 2:**
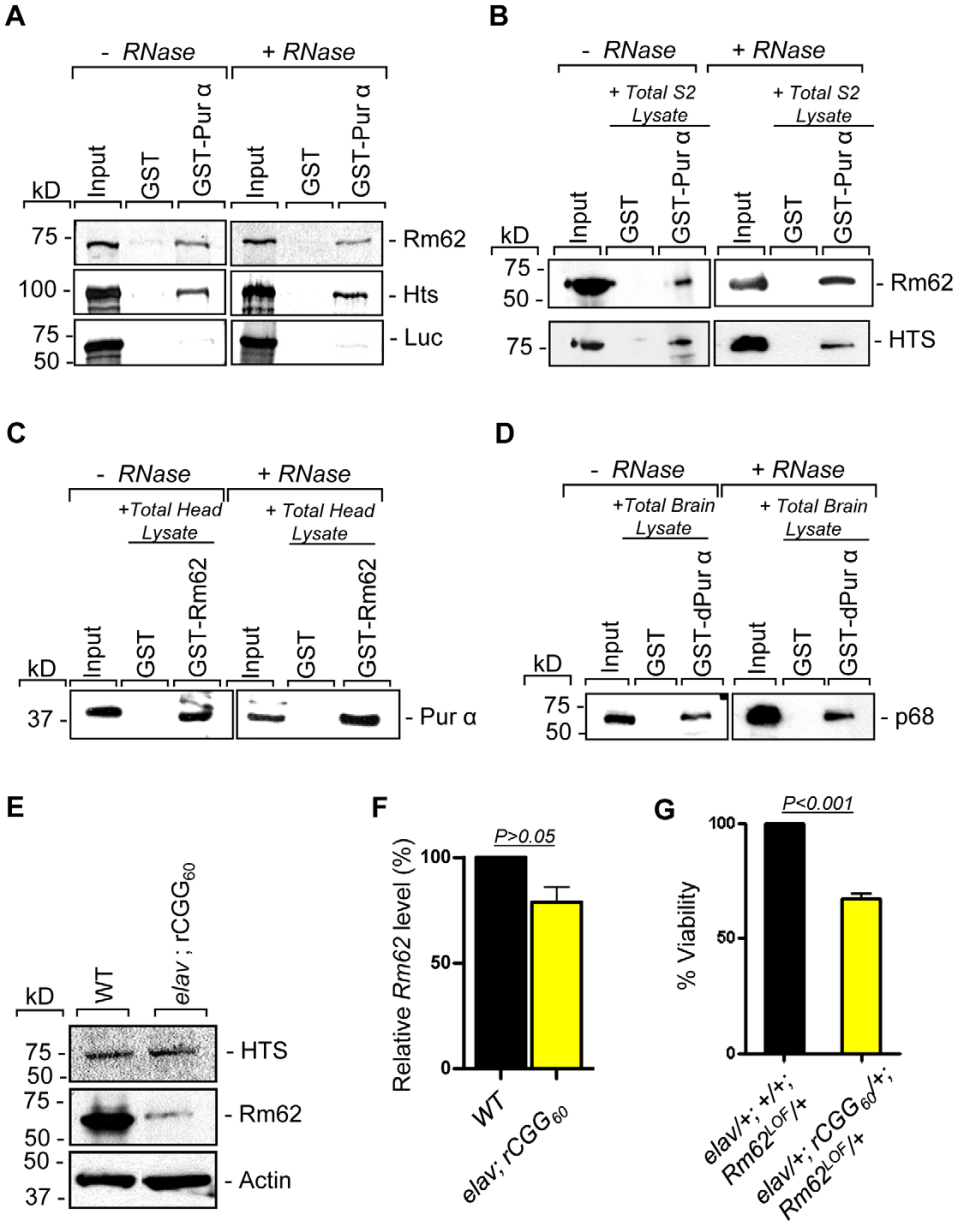
Rm62 and Hts directly interact with Pur α and Fragile X premutation rCGG repeats decrease the expression of Rm62 posttranscriptionally. A. Pull-down assay between GST-tagged dPur α and *in vitro*-translated Rm62 or *in vitro*-translated Hts or luciferase (negative control). Indicated are samples treated or untreated with RNase prior to binding reaction. In the Input lanes, we loaded 25% of the translation products used in a reaction. Both Rm62 and Hts, but not luciferase, interacts with dPur α. B. Western blot shows the interaction between endogenous Rm62 or endogenous Hts and affinity-purified GST-Pur α. Indicated are samples treated or untreated with RNase prior to binding reaction. C. Western blot shows the interaction between affinity-purified GST-Rm62 and endogenous Pur α in RNase untreated and treated samples. D. Western blot shows the interaction between endogenous mammalian p68 and affinity-purified fly GST-Pur α. E. Protein levels of Rm62 and Hts in wild-type and rCGG-expressing flies. Quantitative analysis of Rm62 and Hts protein levels by Western blot on adult head extracts of the following genotypes: wild-type (WT); *elav*; rCGG_60_-expressing flies. Proteins are indicated to the right, corresponding molecular weights to the left. α actin represents a loading control. F. Quantitative analysis of *Rm62* mRNA levels by real-time PCR on total RNA obtained from adult heads of wild-type (WT) and rCGG-expressing flies. Quantification is relative to the housekeeping ribosomal protein 32 (*Rpl32*) mRNA. (E; mean ± SEM n = 3). G. Statistical evaluation of the percent viability displayed by various genotypes: *elav/+; +/+; Rm62^LOF^/+* (Rm62 heterozygous); *elav/+; rCGG_60_-EGFP/+; TM3Sb/+* (Premutation heterozygous), *elav/+; +/+; TM3Sb/+* (Internal control); *elav/+; rCGG_60_-EGFP/+; Rm62^LOF^/+* (interaction). Mean of three data sets was used.

To further determine the nature of biochemical interactions between these two proteins and Pur α we obtained their full-length cDNAs and used them for *in vitro* translation. Either the radiolabeled Rm62 or Hts protein was incubated with either purified recombinant GST-Pur α or GST alone; radiolabeled luciferase was used as a negative control. Both Rm62 and Hts could directly interact with GST-Pur α, but not with GST alone (Figure 2A). We went on to incubate purified recombinant GST-Pur α or GST with fly S2 cell extract. The captured proteins were gel-separated, followed by a Western blot analysis using specific antibodies against Rm62 protein and Hts. Both endogenous Rm62 and Hts could be captured by GST-Pur α (Figure 2B). To further confirm their interactions, we also performed a reverse pull-down experiment in which we incubated recombinant GST-Rm62 or GST with protein lysate from adult fly heads, which express Pur α at much higher levels than S2 cells. The captured proteins were then used for Western blot analysis with a specific antibody against Pur α protein that we developed previously. As shown in Figure 2C, endogenous Pur α could be captured by GST-Rm62 specifically. Lastly, the addition of RNase did not disrupt the interaction between Pur α and Rm62, suggesting that their interaction is RNA-independent. Given that Pur α is a well-conserved RNA-binding protein between *Drosophila* and mammals, we further explored the interaction between purified recombinant GST-Pur α and the mouse ortholog of Pur α-interacting proteins. We performed GST pull-down assays using purified recombinant GST-Pur α and wild-type mouse cerebellar lysates. The captured proteins were used for Western blot analysis with the antibody against the mouse ortholog of Rm62, p68 or DDX5 [28]. As shown in Figure 2D, fly Pur α protein could also interact with p68, suggesting that the interaction between Pur α and Rm62 is conserved through evolution.

### Fragile X premutation rCGG repeats decrease the expression of Rm62 posttranscriptionally

Our earlier studies had shown that ectopic expression of rCGG repeats could sequester the soluble pool of Pur α, preventing its normal function and inducing neuronal apoptosis [17]. Given that Rm62 and Hts could directly interact with Pur α and influence rCGG-mediated neuronal toxicity, we examined their expression in flies expressing fragile X premutation rCGG repeats. In rCGG repeat transgenic flies, the severity of their phenotype depends on both dosage and length of the rCGG repeat. Moderate expression of (CGG)_90_ repeats exclusively in the eyes have an effect on morphology and histology; however, expression of (CGG)_90_ repeats in the neurons leads to lethality at the embryonic stage, preventing analysis at the adult stage [13]. Therefore, we used a shorter repeat length, r(CGG)_60_, which allowed us to examine the expression of these proteins in adults. We performed Western blot analysis using whole-head lysates from both wild-type flies and transgenic flies expressing r(CGG)_60_ repeats in pan-neuronal cells (*Elav-GAL4; UAS-(CGG)_60_-EGFP*). Compared with wild-type flies, we observed a significant decrease of Rm62 protein in rCGG repeat-expressing flies, whereas no change was detected with Hts protein (Figure 2E). We further determined the mRNA level of Rm62 by quantitative RT-PCR and detected no significant difference between wild-type and rCGG repeat-expressing flies (Figure 2F). Furthermore, we generated a genetic combination in which rCGG_60_ repeats were expressed in pan-neuronal cells in the heterozygous background of the *Rm62* mutation. The expression of fragile X premutation rCGG repeats significantly reduced the viability of otherwise viable heterozygous *Rm62* mutant flies, providing further support for a strong genetic interaction between Rm62 and rCGG repeat-mediated neuronal cell death (Figure 2G). Based on these observations, we chose to focus on the role of Rm62 in the context of rCGG-mediated neurodegeneration.

### Fragile X premutation rCGG repeats cause the nuclear accumulation of Hsp70 mRNA

Rm62 is the *Drosophila* ortholog of the p68 RNA helicase. p68 RNA helicase is a prototypical DEAD-box RNA helicase that has been implicated in transcriptional regulation, pre-mRNA splicing, and nucleocytoplasmic shuttling [24], [26], [28]–[30]. In *Drosophila*, Rm62 is reported to play a novel role in RNA export and gene deactivation, particularly *Hsp70* mRNA [31]. Studies from our own and other groups have shown that Hsp70 protein is part of the inclusions induced by fragile X premutation rCGG repeats in both human postmortem brain tissues and FXTAS animal models, including mouse and flies. Like other neurodegenerative disorders, overexpression of *Hsp70* can suppress the neuronal cell death caused by rCGG repeats, suggesting an important role for *Hsp70* in FXTAS pathogenesis [13].

**Figure 3:**
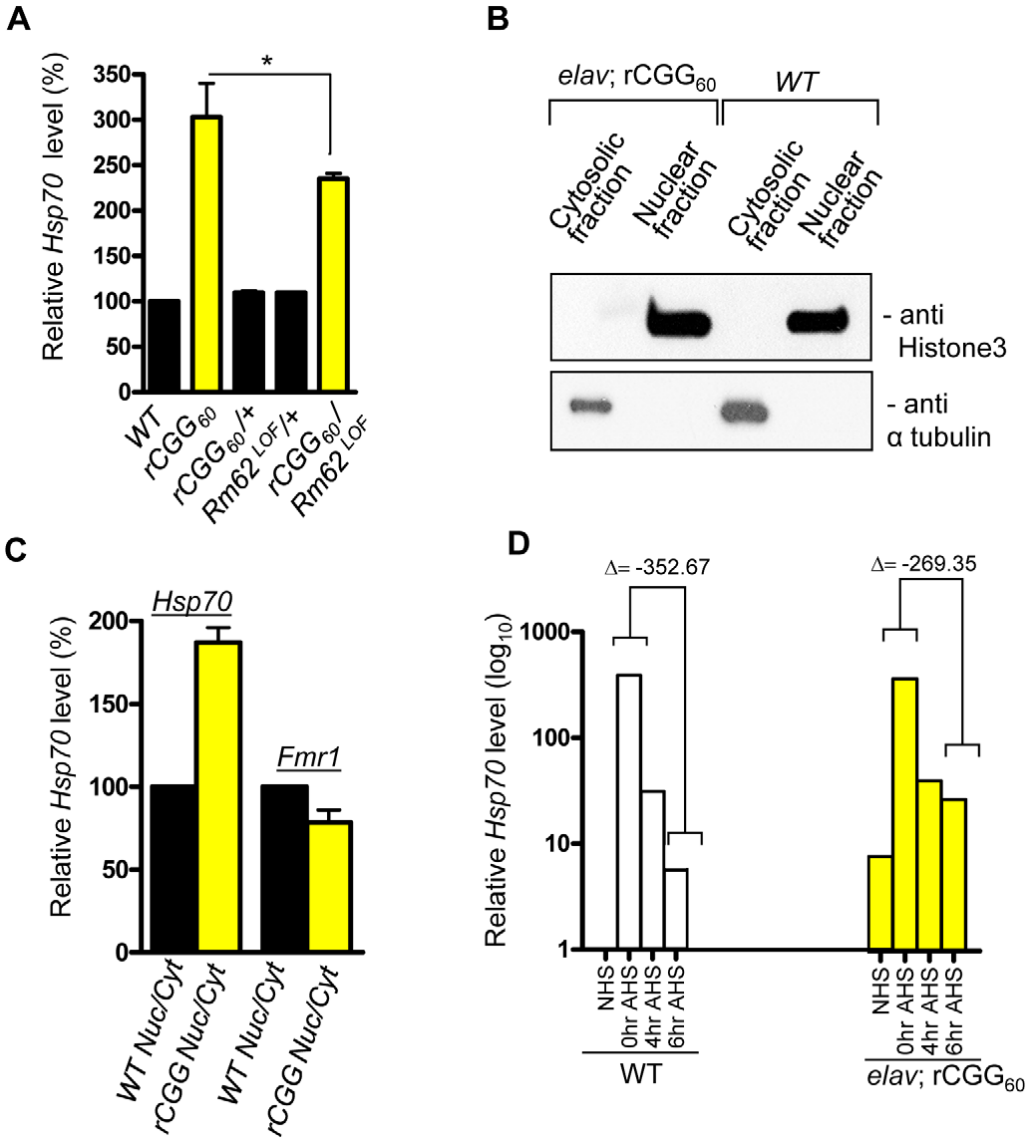
Fragile X premutation rCGG repeats cause the nuclear accumulation of Hsp70 mRNA. A. Quantitative analysis of *Hsp70* mRNA levels by real-time PCR from the adult heads of genotypes: *+/+* (wild-type (WT)); *elav; rCGG_60_* (rCGG-expressing homozygous flies); *elav/+; rCGG_60_/+* (rCGG-heterozygous flies); *Rm62^LOF^/+* (Rm62 mutation heterozygous flies); and *rCGG_60_/+; Rm62^LOF^/+* (interaction). Housekeeping ribosomal protein 32 (*Rpl32*) mRNA was used as an internal control. *: p<0.05 B. Western blot with anti-histone 3 antibody, and α tubulin. C. Quantitative analysis of *Hsp70* mRNA levels by real-time PCR on cytoplasmic and nuclear RNA fractions obtained from adult heads of wild-type (WT) and rCGG-expressing flies. *Rpl32* mRNA was used as control. D. Quantitative analysis of *Hsp70* mRNA levels in total RNA fractions upon heat shock. Both wild-type (WT) and rCGG-expressing flies were heat shocked for 30 min. No heat shock (NHS) represents non-heat shocked controls. Flies were decapitated at the indicated time after heat shock. Heads were collected and total RNA isolated from them. Both WT and rCGG-expressing flies displayed robust expression of *Hsp70* in response to heat shock. After the removal of heat shock, *Hsp70* transcripts declined radically in the WT, whereas in rCGG-expressing flies, *Hsp70* transcripts show prolonged accumulation. Control samples do not display any overt differences in the timing or expression levels of *Hsp70* during the initial response to a short heat shock. Real time against *Fmr1* serves as a control on fractionated samples. The data represent mean ± SEM, n = 3.

Because of the role Rm62 plays in the nuclear export of *Hsp70* mRNA and the significant reduction of Rm62 in transgenic flies expressing fragile X premutation rCGG repeats, we examined the expression and distribution of *Hsp70* mRNA. Total RNA and RNAs from both nuclear and cytoplasmic fractions were isolated from the heads of both wild-type flies and flies expressing rCGG_60_ repeats in pan-neuronal cells. Proteins from both nuclear and cytoplasmic fractions were further analyzed by Western blots using antibody against histone H3, a protein found exclusively in the nucleus. As shown in Figure 3, histone was present in nuclear fractions with minimum cross-contamination in cytoplasmic fractions prepared from both wild-type and rCGG-expressing fly heads. Furthermore, the cytoplasmic fractions were enriched in tubulin, a protein usually present in cytoplasm. We then determined the levels of Hsp70 mRNAs in total RNAs and RNAs from cytoplasmic and nuclear fractions by quantitative RT-PCR. We observed a three-fold increase in *Hsp70* mRNA in the brain total RNAs from flies expressing rCGG_60_ repeats in pan-neuronal cells compared with wild-type flies (Figure 3A). Interestingly, *Hsp70* transcripts were enriched two-fold in the nuclear fraction, while there was no significant change detected in the cytoplasmic fraction from rCGG-expressing flies (Figure 3C). As a negative control, an unrelated transcript, *dFmr1,* was found to be unaltered in either nuclear or cytoplasmic fractions. Furthermore, the elevated expression of *Hsp70* mRNA in flies expressing fragile X premutation rCGG repeats is likely mediated by Rm62, since the partial loss of Rm62 could significantly increase the expression of Hsp70 in rCGG-expressing fly brains.

Previous studies have shown that Rm62 functions in RNA export and is involved in the export of *Hsp70* mRNA following heat shock [31]. The loss of Rm62 will retard RNA export and increase the time required to shut off the transcription of *Hsp70* mRNA [31], so we administered a 30-minute heat shock to both wild-type flies and flies expressing rCGG in pan-neuronal cells (*Elav-GAL4; UAS-(CGG)_60_-EGFP*) and allowed them to recover for up to six hours. During the recovery period (0 to 6 h after heat shock (AHS)), timed samples were removed and analyzed for *Hsp70* transcript production. The control sample showed high levels of *Hsp70* mRNA had accumulated only for a short period following the heat shock, and these levels then began to fall, consistent with previously described kinetics of the heat shock response [32], [33] (Figure 3D). However, in rCGG_60_ transgenic flies, *Hsp70* transcripts persisted for a longer time and could be detected even six hours after the recovery period. This is in contrast to the control samples, where by the end of six hours, *Hsp70* transcripts had dropped to near background levels, reflecting the known rapid shutoff and export of *Hsp70* mRNA in the hours following a 30-minute heat shock (Figure 3D). This observation is consistent with the earlier finding that the loss of Rm62 could delay the clearance of HS-inducible *Hsp70* mRNA. The elevated *Hsp70* level in *Elav*; rCGG transgenic flies prior to heat shock could reflect chronic stress induced by the expression of premutation rCGG repeats. Taken together, these results suggest possible deficits in the nuclear export of *Hsp70* mRNA in the presence of Fragile X premutation rCGG repeats, due to reduced Rm62 levels.

### Nuclear accumulation of selective mRNAs caused by fragile X premutation rCGG repeats

**Figure 4:**
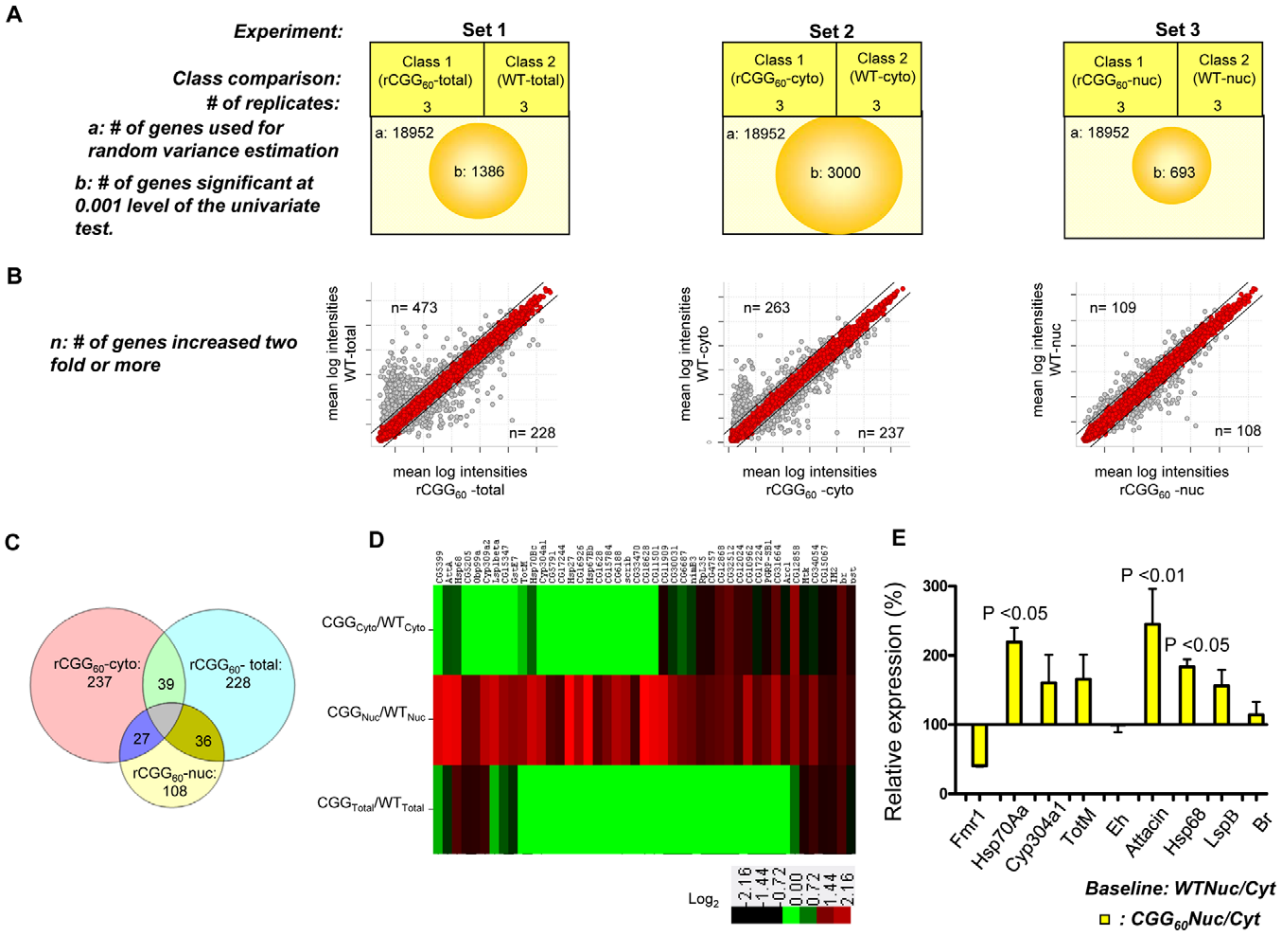
Identification via microarray analyses of selective mRNAs that accumulate in the nucleus as a result of fragile X premutation rCGG repeats. A. The three sets of microarray experiments were carried out in triplicate (three biological replicates) with equal amounts of total RNA obtained from the *Drosophila* heads of control total, nuclear, and cytoplasmic fractions and the corresponding experimental premutation rCGG repeat samples of total, nuclear, and cytoplasmic fractions. Microarray analyses were carried out by identifying significantly changed genes at the 0.001 level of the univariate test. B. Scatter plot of mean log intensities of each sample demonstrating differentially expressed significant genes with fold-change of 2 or more between the classes within a particular set. C. The numbers of unique differentially expressed genes generated by the different cellular compartments of rCGG sample (comparison of total, cytoplasmic, and nuclear fraction) are displayed in Venn diagrams. D. Fold enrichment depicted by ratios between the intensities of normalized log-transformed gene expressions for 45 genes unique to CGG nuclear fractions in various classes is displayed using the Cluster and TreeView programs for the wild-type and premutation rCGG datasets. The fold of the change is indicated on both sides of the scale bar E. Validation of nuclear enrichment by real-time PCR analysis of the selective genes. Real time against *Fmr1* serves as a control. The data represent mean ± SEM, n = 3.

On the premise that rCGG repeat expression in neurons might influence the RNA export pathway, we sought to systematically identify additional RNAs that are altered in their nuclear-cytoplasmic distribution in the presence of fragile X premutation rCGG repeats. To this end, we carried out microarray studies using total RNAs and RNAs isolated from both nuclear and cytoplasmic fractions. A graphical representation of the experimental design and results is shown in Figure 4A. In the first set, total RNA was isolated from age- and sex-matched heads from wild-type (WT) flies and flies expressing rCGG_60_ repeats in neurons, and these were classified as wild-type-total and rCGG_60_-total, respectively. In the second set, RNAs from cytoplasmic fractions were taken from the respective age- and sex-matched heads of wild-type (WT) flies and flies expressing rCGG_60_ repeats in neurons, and these were classified as wild-type-cytoplasmic and rCGG_60_-cytoplasmic, respectively. In the third set, RNAs from nuclear fractions were taken from the respective age- and sex-matched heads of wild-type (WT) flies and flies expressing rCGG_60_ repeats in neurons, and these were classified as wild-type-nuclear and rCGG_60_-nuclear, respectively. To ensure efficient cell fractionation, all the criteria mentioned above had to be met. Also, we used the same microarray procedures and analyses for each sample. Three biological replicates were analyzed for each class using Affymetrix *Drosophila* Genome 2.0 Arrays. The signal intensities of each chip were normalized with RMA algorithms. The correlation coefficients for the three biological replicates were greater than 0.93 in all sets, indicating that the gene expression data obtained in this study were highly reproducible. Genes that discriminate among classes were sorted by p-value of the two-sample t-test (with random variance model). In set 1, out of 18,952 probe sets, 1,386 genes were significant at the 0.001 level of the univariate test. In set 2, out of 18,952 probe sets, 3,000 genes were significant at the 0.001 level of the univariate test. Likewise, in set 3, out of 18,952 probe sets, 693 genes were significant at the 0.001 level of the univariate test. For those genes that expressed differentially between the classes within a particular set, we used a fold-change of two or higher as the filtering criterion, as represented in a typical scatter plot of mean log intensities for each sample (Figure 4B). The number of unique RNAs increased in rCGG-total samples, and rCGG-nuclear as well as rCGG-cytoplasmic fractions were displayed in a Venn diagram. Out of 108 genes/probes that were increased in the rCGG-nuclear class, there were overlaps of 36 and 27 genes, with 228 and 237 genes increased in the rCGG-total and rCGG-cytoplasmic class, respectively. Therefore, the remaining 45 genes unique to the rCGG-nuclear class might represent genes that are only altered in their nuclear cytoplasmic distribution, but without a change in the overall transcriptional level in response to rCGG expression (Figure 4C). The identified genes were then annotated with functional assignments and sorted into gene-related categories using a DAVID functional annotation tool [34]. It is noteworthy that genes involved in both immune response and stress response are enriched in our gene list with statistical values of (15%, p value: 2.77E-07), and (10%, p value: 1.34E-05) respectively. We further compared these 45 genes between the classes within a particular set based on the fold enrichment of their intensities of normalized log-transformed gene expressions. As depicted in Figure 4D, genes showing a remarkable fold change were highlighted. For validation, eight genes were selected, including stress response genes, *Hsps*, *Attacin*, *Turandot*, and *Cytochrome P450* enzyme. As shown in Figure 4E, quantitative RT PCR on RNA derived from total brain lysates of wild-type and rCGG_60_ repeat flies indeed showed significant changes in their expression. *Hsp70Aa* and *Fmr1* were used as positive and negative controls, respectively. These findings confirm that nuclear accumulation in the presence of fragile X premutation rCGG repeats is not limited to the *Hsp70* transcript and also suggest that the nuclear accumulation of these transcripts could be an important factor in the sequelae associated with FXTAS.

### rCGG repeats genetically interact with *small bristles* (*sbr*), the *Drosophila* homolog of the human mRNA export factor *NXF1*

**Figure 5:**
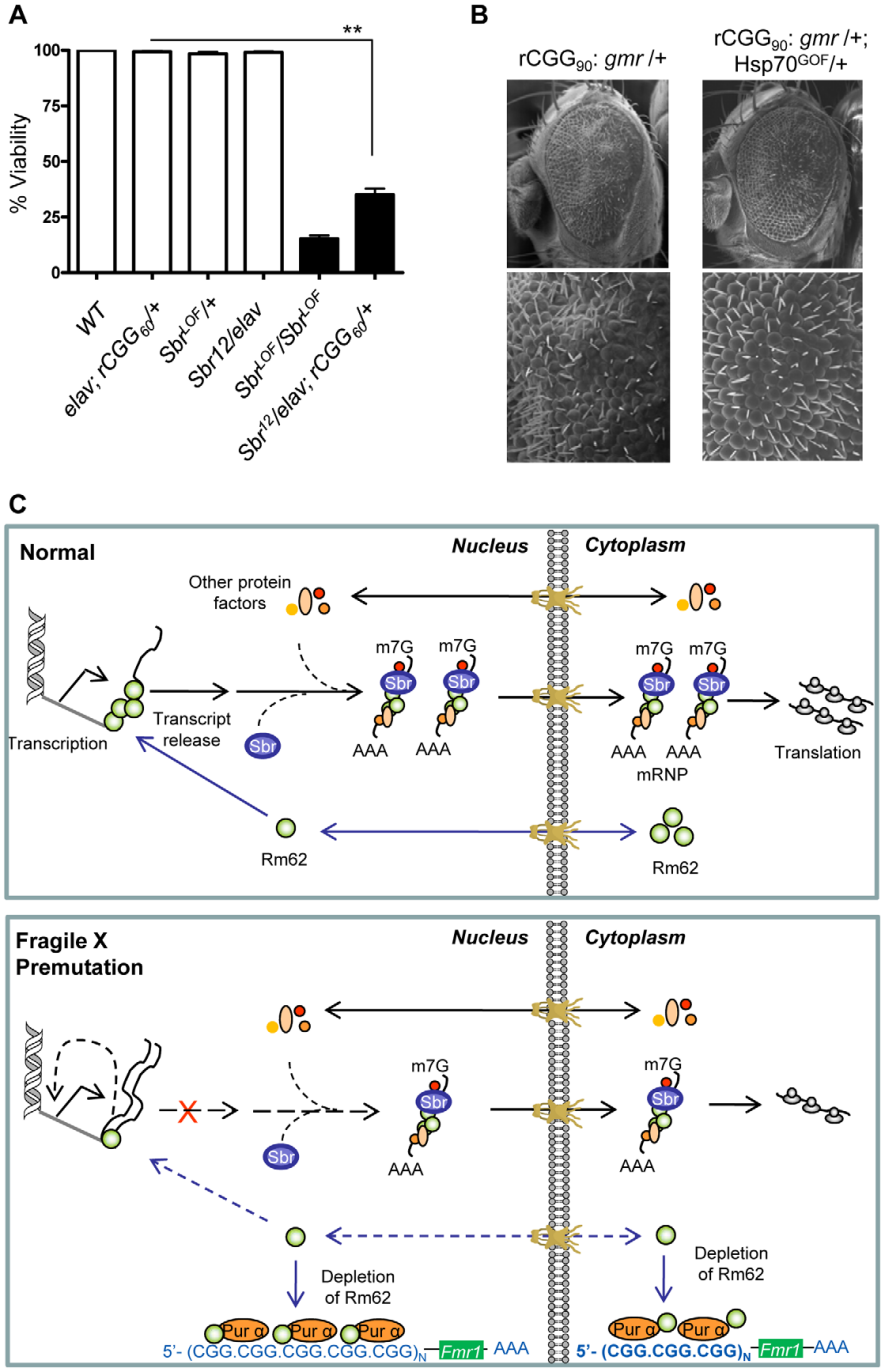
Fragile X premutation rCGG repeats display genetic interaction with the nuclear export factor, small bristles, and the expression of molecular chaperone *Hsp70* suppresses rCGG repeat-mediated neurodegeneration *in vivo*. A. Statistical evaluation of the percent viability displayed by Sbr homozygous and heterozygous flies or premutation heterozygous flies, and their respective interaction experiments *Sbr12^LOF^/elav; rCGG_60_-EGFP/+.* Genotypes: *+/+* (wild-type); *Sbr12^LOF^/Sbr12^LOF^*; *Sbr12^LOF^/+*; *elav/+; rCGG_60_-EGFP/+* and *Sbr12^LOF^/elav; rCGG_60_-EGFP/+* (interaction). Mean of three data sets was used. Error bars indicate SEM. **: p<0.001. B. Shown are SEM pictures of the eyes of adult flies expressing gmr: (CGG)_90_-EGFP/+ only (left), in comparison to *Gmr:* (CGG)_90_-EGFP/+; *Hsp70^GOF^*/+. C. Model representing various interactions involving fragile X premutation rCGG repeats and its potential impact on mRNA nuclear export.

In *Drosophila*, the *small bristles* (*sbr*) and *Hel25E* genes, homologs of the human mRNA export factor *NXF1* and *UASP56* RNA helicase, respectively, are essential for exporting the bulk of *Drosophila* mRNAs, besides heat shock transcripts [35]–[39]. In normal cells, RNA export protein complex shuttles between transcription sites and nuclear pores. Rm62 functions to rapidly remove transcripts from the transcription sites before they begin to move towards nuclear pores. The loss of Rm62 leads to redistribution of export factors within nuclei. Unlike normal cells, where Sbr and other export factors are distributed along the rim of the nucleus and in the nucleoplasm, Rm62 mutations lead to accumulation of Sbr and other export factors in the nucleoplasm. Strong alleles of *sbr* are lethal, and animals bearing weaker *sbr* alleles can survive to adulthood, with a few having abnormally thin thoracic bristles, and females being sterile [39]. *Rm62* mutations phenotypically resemble mutations in *small bristles* (*sbr*) (i.e., lethal and female-sterile) and retain completed mRNAs at their transcription sites [31]. These changes in export factor distribution, as well as the similarities between the *Rm62* and *sbr* mutations, suggest that Rm62 functions upstream or in conjunction with other export factors at sites of gene transcription. If rCGG repeats deplete the Rm62 pool and show biochemical as well as genetic interactions with it, then rCGG repeats might also interact with known RNA export components. To test this hypothesis, male *sbr* heterozygotes were crossed with *elav-GAL4* (X); *rCGG_60_-EGFP*. The F1 progenies were collected every 24 hours for four consecutive days or until culture exhausted. A heterozygous combination of *sbr*/*elav-GAL4; rCGG_60_-EGFP* appears to have delayed development and displayed 30–40 times the lethality of its siblings (p = 0.0035). Another cross between *sbr/y* as well as *Elav* (X); rCGG_60_-EGFP separately with wild-type (*w^1118^)* did not produce any lethality, suggesting that rCGG repeats influence the Sbr-mediated mRNA export pathway (Figure 5A).

## Discussion

Fragile X-associated tremor/ataxia syndrome is a neurodegenerative disorder seen in adult fragile X syndrome premutation carriers. Although several studies, including our own, have proposed a model of RNA-mediated sequestration, in which the sequestration of several RNA-binding proteins (RBP) by rCGG repeat-binding causes cell death and neurodegeneration, the precise mechanisms by which expanded rCGG repeats exert their toxic effects are less understood. Here we report an unexpected nuclear accumulation of specific mRNAs caused by fragile X premutation rCGG repeats, which indicates that a potential deficit in the nuclear export of these mRNAs could contribute to the pathogenesis of FXTAS.

Our earlier studies demonstrated that Pur α and hnRNP A2/B1 are rCGG-repeat-binding proteins and that they could modulate rCGG-mediated neuronal toxicity [17], [18]. In particular, the loss of Pur α in mice is known to cause neurological phenotypes reminiscent of those observed in FXTAS patients [19]. Therefore, to better understand the set of molecular interactions that are dysregulated as a consequence of the sequestration of rCGG repeat-binding proteins (RBPs), we used proteomic analyses to identify the Pur α interactome, which includes proteins involved in different biological pathways. However, whether these interacting proteins could be involved in rCGG repeat-mediated neuronal toxicity was unclear. To address this question, we further tested the roles of Pur α-interacting proteins with the existing fly mutants in rCGG-mediated neurodegeneration. Interestingly, two proteins, Rm62 and Hts, the *Drosophila* orthologs of the p68 RNA helicase and Adducin, respectively, were found to prominently modulate rCGG-mediated neuronal toxicity. These findings suggest that, despite Pur α being involved in multiple biological pathways, only selective pathway(s) altered by fragile X premutation rCGG repeats and the sequestration of Pur α are involved in rCGG-mediated neurodegeneration. Our methods demonstrate the utility and power of combining a proteomic approach with genetic-interaction tests to identify key biological pathway(s) relevant to human diseases.

Rm62/Dmp68 is a member of the DEAD-box family, which includes a large number of proteins that play important roles in almost all aspects of RNA metabolism [28], [29]. Specifically, Rm62/Dmp68 is found to be a multifunctional protein involved in gene transcription, pre-mRNA, pre-rRNA, and pre-miRNA processing, RNAi, and RNA export [21]-[23],[25],[26],[30],[31]. The belief now is that Rm62/Dmp68 may be associated with mRNP export, promoting gene silencing by removing transcripts from their active site of synthesis to promote an inactive heterochromatin configuration [31]. The mammalian ortholog of Rm62/Dmp68 is found to shuttle between cytoplasm and the nucleus [30]. In flies, disruption of its action alters the nuclear distribution of known RNA export components, producing defects similar to those seen in *sbr* mutants, a known component of the RNA export machinery [31]. Given the importance of Rm62/Dmp68 as part of an mRNP complex involved in mRNA transport, it is likely that its depletion or sequestration caused by fragile X premutation rCGG repeats could lead to dysfunction of mRNA transport in the cell and eventual cell death and neuronal toxicity. In line with our observation, other studies have shown that premutation rCGG repeats can biochemically associate with several other DEAD-box RNA helicases, like DDX3, DDX5/p68, and DDX17, as well as colocalize with CGG_60_ RNA aggregates, resulting in the formation of inclusions in cells [40]; this suggests sequestration as a potential mechanism for the Rm62 posttranscriptional reduction.

In our FXTAS fly model, total steady-state levels of *Hsp70* transcripts are increased, which could reflect an initial cellular response to the stress caused by fragile X premutation rCGG repeats. However, we found that most of the increased *Hsp70* mRNAs are trapped in the nucleus, instead of being transported out of the nucleus to produce Hsp70 protein and respond to cell stress. Interestingly, this altered distribution of *Hsp70* transcripts and their delay in turnover is similar to what was seen with *Hsp70* transcripts in the *Rm62/p68* mutation. Therefore, fragile X premutation rCGG repeats could likely produce deficits in the nuclear export of selective transcripts mediated by Rm62, such as *Hsp70* mRNA. This nuclear accumulation will interfere with the neurons' ability to produce sufficient proteins to respond to cellular stress (Figure 5C). To identify additional accumulated mRNAs in the nucleus of neurons expressing fragile X premutation rCGG repeats, we performed gene expression profiling using total RNAs and RNAs from both nuclear and cytoplasmic fractions. Through these analyses, we identified transcripts that were uniquely enriched/restricted in the nuclear fraction. The gene functions of these nuclear-accumulated transcripts cover a variety of biological processes. For example, gene functions like oxidoreductases indicate the involvement of oxidative stress. Likewise, chaperones indicate maintenance of the conformational homeostasis of cellular proteins and RNAs. The cytokine-like interleukin-6 (IL-6) and immune-induced molecules (IM2) regulate part of the immune system. Discovering that these nuclear-enriched transcripts fell into stress, apoptotic, and immunity/defense pathways rather than other biological processes implies that they are either a causative event, or are a consequence of FXTAS pathogenesis. There is a growing body of evidence from both animal and human studies to suggest that stress can intensify inflammation and increase a person's risk for developing various neurological disorders, like Parkinson's disease, multiple sclerosis (MS), and other inflammatory diseases [41], [42]. These studies, along with our findings presented here, suggest that in the presence of toxic RNAs, such as fragile X premutation rCGG repeats, a continuous build up of stress might allow the inflammatory process to run amok and may compromise homeostasis integrity, triggering neuronal cell death/neurodegeneration. Significantly with regard to the data provided above, overexpression of Hsp70 suppresses toxicity of premutation rCGG repeats (Figure 5B). This paradox could be explained by that the nucleus becomes saturated due to induced *Hsp70* transcripts that are forced out of the nucleus to respond to cell stress. While our study provides insights into potential roles of stress response pathways in FXTAS neurodegeneration, in the future, a detailed analysis of accumulated mRNAs will be important to understand their implications in the pathogenesis of FXTAS.

In summary, through systematic proteomic, genetic, and microarray analyses, here we show that the nuclear accumulation of select mRNAs caused by fragile X premutation rCGG repeats may contribute to FXTAS pathogenesis, and the mechanism could be via impaired nuclear export due to the decreased levels of Rm62 seen upon fragile X premutation rCGG expression.

## Materials and Methods

### *Drosophila* strains and genetics

The wild-type strain used was W^118^. All insertions, *Elav-Gal4* (C115) and *Gmr-Gal4*, were from Bloomington Stock Centre (Bloomington, Indiana, USA). Transgenic flies expressing rCGG_90_ and rCGG_60_ repeats were previously generated in the lab (Peng Jin, Emory University School of Medicine, Atlanta, USA). A stable line expressing rCGG_60_ repeats under the control of *Elav-Gal4* was established. Rm62^01084^ and Rm62^CB02119^ fly lines were kindly provided by Allan Spradling. Rm62^(3)3607^, the Rm62 overexpression fly line, was kindly provided by Mani Ramaswami. Other P-element lesions as depicted in Table 2 were from Bloomington. All crosses were grown on standard medium at 25°C, except that the *Elav*; rCCG_60_ line was maintained at room temperature.

### Construction, expression, and purification of GST-Pur α fusion protein

*Drosophila melanogaster* Pur α cDNAs were cloned into vector pGEX-2TK using the respective pairs of primers 5′-ATGTGGATCCATGGAAGATCTCCTCGTG-3′ and 5′- ATGTGAATTCTTAGGACGTGCCATTGAC-3′ with the restriction sites (underlined). Rm62 coding sequence containing a site for ‘Gateway entry clone’ was obtained from cDNA, RE11923, using the respective pairs of primers 5′ CACC ATGCTTAAGCTTGTGCAATACATAG and GTCGAAGCGCGAGTGTCT with the entry site (underlined). Rm62 CDS was subsequently cloned in pDEST24 Gateway expression vector. The open reading frame of both fusion genes was confirmed by sequencing. Both pGEX-Pur α and pGEX-2TK were individually transformed into the *E. coli* expression strain BL21 (DE3). Overexpression of pGEX-Pur α or pGEX-2TK alone was induced by 16 µM IPTG for 7 h at room temperature. Overexpression of pDEST-Rm62 or pDEST24 alone was induced by 0.2% L-arabinose for 8 h at room temperature. Bacteria expressing corresponding proteins were lysed in B-PER Protein Extraction Reagents (Pierce, Thermo Scientific). Purification of GST-tagged Pur α and GST alone was carried out by affinity chromatography using glutathione immobilized to a matrix sepharose, which were treated with both DNase and RNase before being used for capture experiment.

### Capture of the interacting proteins with dPur α

The GST-fusion protein affinity purification was performed based on a previously reported procedure [43]. Total protein lysates were obtained from adult wild-type fly heads using 50 mM Hepes, pH 7.2, 0.6 M NaCl, 15% glycerol, 20 mM CHAPS, 0.1% Triton, 1 mM DTT, 1 mM EGTA, 1 mM EDTA, and protease inhibitors (Complete Mini, Roche). Ten milliliters of each extract (10 mg/ml) were dialyzed against 1L of dialysis buffer (20 mM Hepes, pH 7.2, 0.1 M NaCl, 5% glycerol, 1 mM DTT, 1 mM EGTA, 1 mM EDTA) twice to reduce the concentration of salt and detergent. After dialysis, 1 mM of DTT and protease inhibitors were added and centrifuged at 500,000 g for 2 h (60K on SW 60 Ti rotor). Supernatant was collected and run through columns of glutathione beads (200 µl) previously equilibrated with the purified GST-Pur α or GST alone. After washing, the sequential elution of bound proteins was performed with the following buffers: Buffer 1 (20 mM Hepes, pH 7.2, 0.2 M to NaCl, 15% glycerol, 0.1% Triton, 1 mM DTT, 1 mM EGTA, 1 mM EDTA); Buffer 2 (20 mM Hepes, pH 7.2, 0.6 M NaCl, 15% glycerol, 0.5% Triton, 1 mM DTT, 1 mM EGTA, 1 mM EDTA); and Buffer 3 (20 mM Hepes, pH 7.2, 2 M NaCl, 15% glycerol, 2% Triton, 1 mM DTT, 1 mM EGTA, 1 mM EDTA), respectively. Eluants were combined, precipitated with cold acetone, washed, and resuspended in protein loading buffer (2% SDS, 60 mM Tris-HCl pH 6.8, 100 mM DTT, 10% glycerol, 0.01% bromophenol blue) for mass spectrometric sequencing analyses.

### Liquid chromatography-tandem mass spectrometry (LC-MS/MS)

Protein identification was performed on an optimized LC-MS/MS platform as described previously [44]. The captured proteins by affinity-purified GST-Pur α and GST alone were separated on a 10% SDS gel, and each lane was cut into multiple pieces, followed by in-gel tryptic digestion. Peptide samples were dissolved and analyzed by nanoscale reverse phase chromatography coupled with an LTQ-Orbitrap mass spectrometer (Thermo Finnigan). All MS/MS spectra were searched against a composite database containing the NCBI mouse or fly reference database and a decoy database. The dataset was filtered to reduce the protein false-discovery rate to less than 1% [45]. To further eliminate false identifications, proteins matched by a single peptide were manually validated.

### GST pull-down assay

Full-length cDNA of Rm62 (RE11923) and Hts (SD02552) were obtained from DGRC, Indiana, USA. Both cDNAs were separately used for *in vitro* transcription–translation in rabbit reticulocyte lysate in the presence of [^35^S] methionine, according to the manufacturer's instructions (Promega). *In vitro*-translated proteins were mixed with purified GST-Pur α or GST alone. The pull-down assay was performed in the presence of 150 mM NaCl. Total cell lysate obtained from S2 cells or from adult fly heads or from mouse cerebellum was incubated overnight with Pur α-GST or Rm62-GST or GST only. Beads were extensively washed in lysis buffer, directly boiled in SDS-PAGE loading buffer, and subjected to SDS-PAGE analysis. 1X proteinase inhibitor was maintained at each step. For the RNAse treatment, a potent RNase Cocktail, a mixture of RNase A (500 U/ml) and RNase T1 (20,000 U/ml) (Ambion, Applied Biosystems) was used in a dilution of 2.5 µl/50 µl of sample.

### Protein and RNA isolation

Heads from adult flies expressing either fragile X premutation rCGG repeats (*Elav-GAL4; UAS-(CGG)_60_-EGFP*) or WT were collected and homogenized in 200 µL of ice-cold lysis buffer (10 mM Tris, pH 7.4, 150 mM NaCl, 30 mM EDTA, 0.5% Triton X-100) with 2X complete protease inhibitors (Roche) and kept on ice for 30 min. Supernatant of 12,000 g was collected, and the amount of total protein was determined by Bradford assay.

S2 cells were cultured in Schneider cell medium (Gibco BRL/Invitrogen) + 10% FBS. Total cell extracts were prepared by lysing S2 cells in buffer (150 mM NaCl, 20 mM Tris-Hcl pH 7.4, 5 mM MgCl2, 0.4% Triton X-100, protease inhibitor (complete mini, Roche)), followed by incubation on ice for 30 min. The supernatant of 12,000 g was collected, and the amount of total protein was determined by Bradford assay.

For the RNA isolation, adult heads of the required genotype were mashed in Trizol (Gibco BRL Life Technologies) using plastic kontes. After precipitation, RNA was further cleaned with RNeasy (QIAGEN).

### Western blot analysis

We used standard Western blotting techniques with Tris Buffer, pH 8.3. For the detection of Rm62, transfer was carried in CAPS buffer, pH 11. Antibodies were used at the following concentrations: rat anti-Rm62 (1∶800; kind gift from Elissa Lei, Bethesda, MD, USA); Hts supernatant (1∶5; Hybridoma); rabbit anti-Pur α (generated previously in the lab; mMad1 (kind gift from Frances Fuller-Pace); histone H3 (1∶5000, Abcam);α tubulin (1∶1000, Abcam); goat anti-mouse HRP (1∶5000); Sigma); goat anti-rabbit HRP (1∶5000; Sigma); and goat anti-rat HRP (1∶5000; The Jackson Laboratory).

### RT-PCR and quantitative RT-PCRs

For RT-PCR, RNA was reverse-transcribed with random primers using the high-capacity cDNA reverse transcription kit (Applied Biosystems) according to the manufacturer's instructions. Real-time PCR was performed with gene-specific primers and Power SYBR Green PCR Master Mix (Applied Biosystems) using 7500 Fast Real-Time PCR system (Applied Biosystems). Primers were designed using Primer Express software (Applied Biosystems) or the commercially available QuantiTect primers from Qiagen. For *Rm62* the following primer combination was used: CGAGAAGCTAATCAGGAAATCAATC and ACCGTCGTAGCGCGAGTT. For *Hsp70* the following primer combination was used: CTCGTCGGCGGATCCA and GGAAGAACTCCTGCAGCAGACT. For *Fmr1* the following primer combination was used: CTCGTCGGCCCAGCA and GGAAGAACTTCGAGCAGACT. For the endogenous control, QuantiTect Dm_RpL32_1_SG (Qiagen) was used according to the manufacturer's instructions.

### Preparation of nuclear and cytoplasmic fractionations

Flies of the required genotypes were snap-frozen in dry ice and then decapitated. Heads were collected and crushed to powder using motorized plastic kontes in dry ice. Powdered heads were resuspended in cell fractionation buffer from the PARIS kit (Ambion) and kept on ice for 30 min to 1 h, followed by low-speed centrifugation of 500 g to pellet nuclei together with debris. Carefully, supernatant was collected as the cytoplasmic fraction, and the pellet as nuclear fraction. The pellet/nuclear fraction was washed again with cell fractionation buffer to remove any lingering supernatant. Protein and RNA were isolated from the same homogenate using the PARIS kit (Ambion) according to the manufacturer's instructions. RNA was quantified, and RT-PCR was performed using ABI (Invitrogen, Carlsbad, CA) according to the manufacturer's instructions.

### Microscopy and immunohistochemistry

Genetic interactions based on the rough eye enhancement or suppression were initially assessed under the dissection microscope for more than 50 flies. To confirm suppression and to document phenotypes, we compared 6-10 SEM images of eyes from experimental animals with those of “average” (rCGG)_90_. For scanning electron microscopy (SEM) images, whole flies were serially dehydrated in ethanol, dried with hexamethyldisilazane (Sigma-Aldrich), and analyzed with an ISI DS-130 LaB6 SEM/STEM microscope.

### Microarray analyses

cRNA amplification and fluorescence labeling was performed according to the supplier's instructions using the Affymetrix 3′IVT kit (Affymetrix Technologies). The labeled target was combined and allowed to hybridize to probes on the *Drosophila* genome 2.0 array according to instructions. The arrays were washed using the Midi _euk2v3 fluidics protocol and scanned using the Microarray Scanner laser-based detection system. All normalizations were performed using default settings.

Image data were quantified using the Affymetrix expression console. All analysis was performed using Bayesian infinite mixture models as implemented in the BBR software, version 3.8.1 (http://linus.nci.nih.gov/BRB-ArrayTools.html), an integrated package for the visualization and statistical analysis of DNA microarray gene expression data. Gene expression data were normalized using the robust multi-array average (RMA) statistical algorithms built in BRB. All filtering parameters were turned off. Class comparison and cluster analysis was performed using the Bayesian infinite mixture models as implemented in the BBR software. Heat map was generated by clustering genes and arrays with complete linkage uncentered correlation using Cluster 3 and Java TreeView [46].

Significantly differentially expressed genes were annotated with functional assignments to help determine which gene categories were enriched with differentially expressed genes. Genes were annotated and biological processes analyzed using the Database for Annotation, Visualization and Integrated Discovery (DAVID) (http://david.abcc.ncifcrf.gov/) [34].

### Statistical method

Statistical analysis was performed using ANOVA with post-hoc t-tests (two samples assuming equal variances) using GraphPad Prism software to determine significance, and indicated *p* values. All data are shown as mean with standard error of mean (mean ± SEM).
